# Cosolvent Dimethyl Sulfoxide Influences Protein–Ligand Binding Kinetics via Solvent Viscosity Effects: Revealing the Success Rate of Complex Formation Following Diffusive Protein–Ligand Encounter

**DOI:** 10.1021/acs.biochem.2c00507

**Published:** 2022-12-21

**Authors:** Sven Wernersson, Simon Birgersson, Mikael Akke

**Affiliations:** Division of Biophysical Chemistry, Center for Molecular Protein Science, Department of Chemistry, Lund University, P.O. Box 124, SE-221 00Lund, Sweden

## Abstract

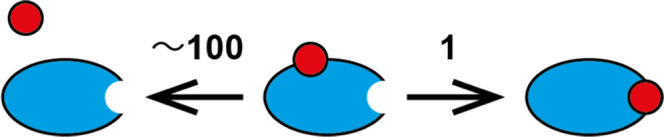

Protein–ligand-exchange kinetics determines the duration of biochemical signals and consequently plays an important role in drug design. Binding studies commonly require solubilization of designed ligands in solvents such as dimethyl sulfoxide (DMSO), resulting in residual amounts of DMSO following titration of solubilized ligands into aqueous protein samples. Therefore, it is critical to establish whether DMSO influences protein–ligand binding. Here, we address the general and indirect effect of DMSO on protein–ligand binding caused by solvent viscosity, which is strongly dependent on the relative concentrations of DMSO and water. As a model system, we studied the binding of a drug-like ligand to the carbohydrate recognition domain of galectin-3 in the presence of variable amounts of DMSO. We used isothermal titration calorimetry to characterize binding thermodynamics and ^15^N NMR relaxation to monitor kinetics. The binding enthalpy is not affected, but we observe a subtle trend of increasingly unfavorable entropy of binding, and consequently decreased affinity, with increasing DMSO concentration. The increasing concentration of DMSO results in a reduced association rate of binding, while the dissociation rate is less affected. The observed association rate is inversely proportional to the viscosity of the DMSO–water mixture, as expected from theory, but significantly reduced from the diffusion-controlled limit. By comparing the viscosity dependence of the observed association rate with that of the theoretical diffusion-controlled association rate, we estimate the success rate of productive complex formation following an initial encounter of proteins and ligands, showing that only one out of several hundred binding “attempts” are successful.

## Introduction

Understanding molecular recognition between proteins and ligands is central to physical and life sciences and is a key aspect of drug design. Ligand binding kinetics has come into focus in the last decade because of its role in determining the lifetime of the ligand–protein complex, which in turn governs the duration of a biochemical signal or its inhibition.^1−5^ Furthermore, the lifetime of the complex, which is equal to the inverse of the dissociation (off-) rate constant, is often found to be a superior predictor of in vivo efficacy compared to the equilibrium binding constant.^5−7^ These observations have spawned initiatives to optimize binding kinetics.^5^ We have previously investigated the binding kinetics of two series of congeneric ligands designed to inhibit the carbohydrate recognition domain of galectin-3 (Gal3C).^8^ Notably, the two ligand series showed different linear free-energy relationships between the off-rate constants and the equilibrium affinity, suggesting that the ligand structure affects the position of the transition state along the generalized reaction coordinate of the binding process.^8^ Furthermore, previous studies have indicated that the association (on-) rate depends on the nature of the initial encounter complex.^9^

Galectin-3 is a member of the galectin family of carbohydrate binding proteins, which have a highly conserved carbohydrate recognition domain. Gal3C is implicated in numerous cellular functions, including cell differentiation, cell cycle regulation, and apoptosis, making it a target for treatment of inflammation and cancer.^10−12^ The ligand binding site in Gal3C is located in a shallow and water-exposed groove across a six-stranded β-sheet, where a number of hydrophilic residues are poised to coordinate ligand oxygen atoms arranged in a sugar-like pattern.^13^ The relatively high solubility of natural galectin ligands in an aqueous solution results in low affinity and makes it challenging to design high-affinity synthetic ligands. Nonetheless, compounds with nanomolar dissociation constants have been developed toward Gal3C by successfully increasing the hydrophobicity while maintaining polar interactions with the canonical ligand-coordinating protein side chains.^14,15^ In most cases, the increased hydrophobicity of these ligands reduces their solubility in water.

Indeed, designed organic compounds targeting proteins are commonly poorly soluble in water. For this reason, in vitro binding studies involving such compounds often require mixed solvents to achieve the desired solubility. Dimethyl sulfoxide (DMSO) is one of the most commonly used organic solvents because it is completely miscible with water and has low chemical reactivity. Ligands can thus be solubilized at high concentrations in DMSO and subsequently be added to an aqueous protein solution to determine the binding constant by titration. The resulting protein–ligand solution typically contains a few percent DMSO, and rarely more than ten percent, which is not expected to affect the protein structure or stability to any greater extent,^16−18^ although contrasting results have been reported in some cases,^19^ and cell-based screening methods often have a DMSO tolerance of less than 2%.^20^ Long-lived interactions between DMSO and proteins that can perturb the protein structure and function seem to require suitable binding pockets or clefts,^21^ whereas transient interactions with protein surface are not sufficient in this regard.^22,23^ However, the viscosity of DMSO–water mixtures is strongly dependent on the amount of DMSO present, particularly in the dilute regime, where the viscosity increases linearly by a factor of 3 as the volume fraction of DMSO increases to 20%.^24−26^ Thus, it is conceivable that DMSO indirectly affects protein function via the change in viscosity. We have previously demonstrated that this effect on viscosity, when uncorrected for, can lead to erroneous conclusions in studies of protein conformational dynamics.^22^ Given the importance of characterizing binding kinetics, it is critical to understand how cosolvents might affect the kinetic rate constants.

Here, we address the question whether DMSO might influence the binding affinity and kinetics. As a model system, we use Gal3C and a designed, drug-like compound with micromolar affinity and sufficiently high solubility in water such that it does not require the addition of DMSO to reach the concentrations used in our in vitro binding studies.^8^ We observe subtle variation in binding affinity, which is driven by changes in entropy. Furthermore, the kinetic on-rate for binding varies with the DMSO concentration in the manner expected from the viscosity of the DMSO–water mixtures. In contrast, the off-rate for binding shows a weaker dependence on viscosity. We take advantage of the linear variation of on-rate with the inverse of viscosity to determine the success rate of ligand binding following protein–ligand encounter. We find that each diffusive encounter between ligands and proteins has less than 1% chance of forming a productive complex before the encounter complex dissociates.

## Materials and Methods

### Protein Expression and Purification

The C-terminal domain of Galectin-3 (UniProt accession number P17931) was expressed and purified following published protocols,^27,28^ yielding a protein stock solution of 16 mg/mL in buffer consisting of 10 mM Na_2_HPO_4_, 1.8 mM KH_2_PO_4_, 140 mM NaCl, 2.7 mM KCl, pH 7.3, 2 mM ethylenediaminetetraacetic acid (EDTA), 4 mM tris(2-carboxyethyl)phosphine hydrochloride (TCEP), and 150 mM lactose. The protein stock solution was stored at 278 K.

### Ligand Synthesis and Purification

The ligand *ortho*-fluoro-phenyltriazolyl-galactosylthiolglucoside has been described before and shown to be fully water soluble.^8,29^

### Isothermal Titration Calorimetry

Gal3C was prepared by extensive dialysis against 5 mM 4-(2-hydroxyethyl)-1-piperazinethanesulfonic acid (HEPES) buffer pH 7.4 to remove all lactose, followed by centrifugation at 14,000 rpm to remove any aggregates. The protein concentration in each sample was determined using UV absorbance.^28^ Ligand and protein solutions at specified DMSO concentrations were prepared using a 50% stock solution of DMSO in 5 mM HEPES buffer to yield samples with 2, 6, or 10% DMSO and pH 7.4. The protein concentration was 0.74 mM.

ITC experiments were performed on a MicroCal PEAQ–ITC instrument (Malvern Panalytical Ltd.) at a temperature of 301 K by titrating the ligand at a concentration of 1.5 mM into the cell containing the protein at a concentration of 150 μM. The DMSO concentrations in the cell and the syringe were carefully matched to minimize the heat of dilution. Three replicate experiments were performed for each condition (DMSO concentration), with an initial injection volume of 0.4 μL, followed by nine injections of 4 μL each using a spinning speed of 750 rpm, a reference power of 10 μcal/s, and a duration of 0.8 s for the first injection and 8 s for the subsequent injections. Each of the triplicate experiments was carried out by concatenating two runs, each with 10 injections, as described above, to generate a single thermogram comprising 18 injections (after subtracting the two initial injections). Additional measurements were carried out for the 0 and 6% DMSO samples in the same manner, but with a factor of 2 lower injection volume to acquire a greater number of datapoints in the initial phase of the titrations and thereby improve the definition of the baseline; these runs were performed in duplicate.

Individual thermograms were concatenated and corrected for baseline differences. Peak integration was done using NITPIC.^30^ A single-site binding model was fitted simultaneously to the three titration curves using SEDPHAT^31^ to yield the binding enthalpy (Δ*H*°), fraction of binding-competent protein (*n*), and dissociation constant (*K*_d_).
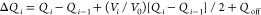
1where *V*_*i*_ is the volume of the *i*th injection, *V*_0_ is the cell volume, *Q*_off_ is an offset parameter that accounts for the heat of mixing, and *Q*_i_ is the heat function following the *i*th injection
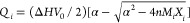
2where α = *nP*_*i*_ + *L*_*i*_ + *K*_d_ and *P*_*i*_ and *L*_*i*_ are the total concentrations of the protein and ligand, respectively, in the cell at any given point of titration. The free energy and entropy of binding were subsequently determined using the relationships Δ*G*° = *RT* ln(*K*_d_) and −*T*Δ*S*° = Δ*G*° – Δ*H*°. Although SEDPHAT reports asymmetric error estimates, the present analysis resulted in nearly symmetric errors, which we report as the average of the upper and lower error bounds. Graphical representations of thermograms and isotherms were prepared using GUSSI.^31^

### NMR Sample Preparation

Samples were prepared for NMR with a target saturation of 95% in mind, resulting in samples of 0.4 mM ^15^N-labelled Gal3C, 0.42 mM ligand, and with 0, 2, 6, or 10% DMSO (v/v) in 5 mM HEPES buffer pH 7.5. Thus, the protein concentration in the NMR samples is only a factor of 2.7 greater than in the ITC experiments, making the conditions highly similar.

### NMR Relaxation Dispersion Experiments

Backbone amide ^15^N CPMG relaxation dispersion experiments were performed at static magnetic field strengths of 11.7 T, using a Varian/Agilent VNMRS Direct Drive spectrometer equipped with a room-temperature triple-resonance probe, and 14.1 T, using a Bruker Avance NEO spectrometer equipped with a 5 mm HPCN QCI cryoprobe. All experiments were performed at a temperature of 301 K. Temperature calibration was performed prior to each series of relaxation experiments using a neat protonated methanol sample.^32,33^ The sample pH was adjusted immediately before each series of relaxation experiments and checked after each series to ensure that the sample pH had not drifted. Constant time relaxation-compensated CPMG experiments^34,35^ were performed at 11.7 T using a 40 ms constant time relaxation period with CPMG refocusing frequencies, ν_cpmg_, of (3 × 0, 50, 100, 150, 200, 300, 400, 500, 650, 800, and 950) Hz and interleaved sampling of *t*_1_ points with different values of ν_cpmg_. Experiments were acquired with a 2 s recovery delay, 80 scans for each 2D plane, and spectral windows in (*t*_1_, *t*_2_) of (1620, 7023) Hz, sampled over (128, 2210) points. Experiments performed at 14.1 T used ν_cpmg_ = (2 × 0, 50, 100, 2 × 300, 400, 500, 600, 700, 800, 900, 1000, and 1100) Hz, 2 s recovery delay, 24 scans for each 2D plane, and spectral windows in (*t*_1_, *t*_2_) of (2129, 9615) Hz, sampled over (128, 2306) points.

### NMR Relaxation Data Analysis

All spectra were processed using NMRPipe.^36^ The processing protocol included squared cosine-bell window functions in both dimensions, a solvent filter, zero-filling to twice the size rounded to the nearest power of 2, and polynomial baseline correction in the direct dimension. Linear prediction to twice the number of datapoints was applied in the indirect dimension. Peak volumes were extracted using PINT, which employs line shape fitting to resolve overlapped peaks.^37,38^ Peak intensities were evaluated using a weighted sum of Lorentzian and Gaussian line shapes. The uncertainties of the fitted peak volumes were estimated from the base plane noise.

The relaxation dispersion data were analyzed using in-house Matlab scripts. Relaxation dispersion curves were fitted to the Carver–Richards two-state exchange model^39,40^
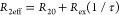
3in which
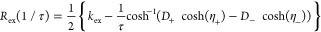
4
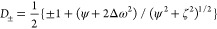
5
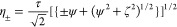
6and ψ = *k*_ex_^2^ – Δω^2^, ζ = −2Δω*k*_ex_(1 – 2*p*_F_); *k*_ex_ = *k*_1_ + *k*_–1_ is the sum of the forward and reverse rate constants corresponding to *k*_on_[*L*] and *k*_off_ in the present case; Δω is the chemical shift difference between the exchanging free and ligand-bound states; *R*_20_ is the average limiting value of the relaxation rate constant for processes other than chemical exchange; *p*_F_ is the population of the (less populated) free state, which is related to the bound state by *p*_F_ = 1 – *p*_B_; and τ = 1/(2 ν_cpmg_) is the spacing between refocusing pulses in the CPMG pulse train.

In fitting the exchange model to the data, we fixed Δω to the value derived from the chemical shifts measured in the spectra of free and fully saturated states. We performed two separate sets of fits in which *p*_F_ was either included as a free parameter of the fit or fixed at the values calculated from *K*_d_ and the total concentrations of protein and ligand in the sample. The statistical significance of each fit was assessed by also fitting the data to a constant *R*_20_ value (i.e., modeling a flat dispersion profile, indicating the absence of exchange), and the *F*-test was used to discriminate between models by rejecting the simpler model at the level *p* < 0.001. Errors in the fitted parameters were estimated from 1000 synthetic data sets created using Monte Carlo simulations.^9^

## Theory

The diffusion-controlled on-rate constant describing the encounter of two spherical molecules, ligand (*L*) and protein (*P*), is given by^41,42^
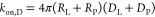
7where *R*_L_ + *R*_P_ is the contact distance between the centers of the two spheres (sum of the radii of L and P) and *D*_L_ + *D*_P_ is the relative diffusion coefficient of the ligand–protein pair. The diffusion coefficient is given by the Stokes–Einstein equation, *D*_*i*_ = *k*_B_*T*/(6π*R*_*i*_η), where η is the solvent viscosity. Thus, *k*_on,D_ can be expressed as
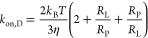
8

For the system studied here, the radii are approximately *R*_L_ = 6 Å and *R*_P_ = 16 Å, yielding an approximate value of *k*_on,D_ ≈ 3.36 *k*_B_*T*/η.

In reality, each encounter does not lead to productive binding because the encounter complex can dissociate before the ligand has had time to diffuse across the protein surface into the binding site. Thus, the binding process involves a pre-equilibrium of the encounter complex and a diffusive search over the protein surface from the point of first contact to the binding site^41^
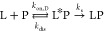
where L*P and LP denote the encounter complex and final complex, respectively, *k*_dis_ is the rate constant for dissociation of the encounter complex, and *k*_s_ is the rate constant for the search process. The effective on-rate constant is then given by
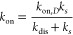
9

Assuming that viscosity mainly affects *k*_on,D_ and much less so *k*_dis_ and *k*_s_, the slope of *k*_on_ versus 1/η can be compared with the slope expected for a diffusion-controlled reaction, that is, the slope of *k*_on,D_ versus 1/η as given in eq 8, to yield the ratio *k*_s_/*k*_dis_ = ρ, which provides an estimate of the “success” rate of productive complex formation in the binding site following the initial encounter between the protein and ligand
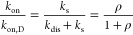
10so that ρ = (*k*_on_/*k*_on,D_)/(1 – *k*_on_/*k*_on,D_). Thus, if *k*_on_ ≪ *k*_on,D_, then ρ ≈ (*k*_on_/*k*_on,D_), which is shown to be valid in the present case (see the Results section).

## Results and Discussion

The primary question we address in this study is whether the commonly used cosolvent DMSO changes the ligand binding equilibrium and kinetics via its effect on viscosity. Second, we use the viscosity dependencies of the observed on-rate constant for ligand binding and the calculated diffusion-controlled on-rate constant to determine the ratio *k*_s_/*k*_dis_. This issue could in principle be addressed using a variety of viscogens. However, given that the natural function of Gal3C is to bind sugar-like molecules,^13^ many common viscogens, such as sucrose and polyethylene glycol, are not suitable for this purpose. Thus, we base our study entirely on samples containing variable concentrations of DMSO. We performed ITC experiments and NMR relaxation dispersion experiments at four different sample conditions: without DMSO and with three different concentrations of DMSO, 2% (v/v), 6%, and 10%. While we used samples of relatively low ion concentration, NMR relaxation dispersion experiments can also in general be conducted at physiological salt concentrations.^43^ NMR relaxation dispersion measurements are capable of characterizing slow to intermediate exchange rates, which correspond to off-rate constants broadly in the range of 1–100 s^–1^.

### ITC Experiments Reveal Subtle Effects of DMSO on Binding Affinity

**Figure 1:**
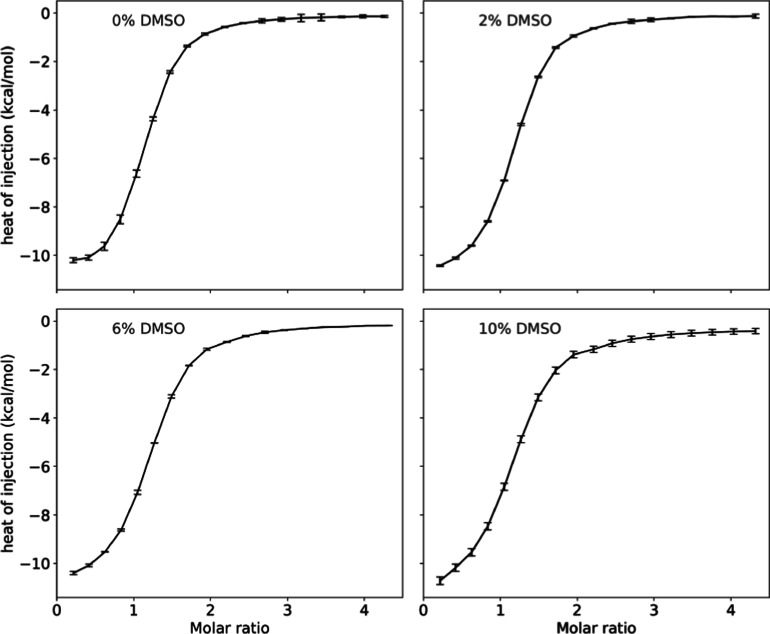
Representative isotherms from ITC measurements of ligand binding to Gal3C at four DMSO concentrations: 0%, 2%, 6%, and 10% v/v. The individual datapoints show the heat of each injection point with error bars indicating the estimated uncertainty in each measurement. The curves represent the best-fit single-site binding model yielding the binding enthalpy (Δ*H*°), fraction of binding-competent protein (*n*), and dissociation constant (*K*_d_), from which Δ*G*° = *RT* ln(*K*_d_) and −*T*Δ*S*° = Δ*G*° – Δ*H*° are calculated. Figure S1 shows all binding isotherms.

We used ITC to determine the thermodynamic fingerprint of ligand binding to Gal3C (Figures 1 and S1 and Table 1). The enthalpy of binding does not depend on the DMSO concentration, in agreement with previous work showing that DMSO does not interact with the ligand binding site of Gal3C.^22^ The entropy of binding also does not show a statistically significant variation with DMSO concentration, although there is a modest trend toward increasingly unfavorable entropy, resulting in a slight increase in *K*_d_ with increasing DMSO concentration. Given that DMSO does not inhibit the binding site, the observed effect of DMSO on the binding affinity must therefore arise from indirect effects.

**Table 1:** Thermodynamics of Ligand Binding

*c*(DMSO) (v/v %)	–Δ*H°*(kJ/mol)	–*T*Δ*S*° (kJ/mol)	–Δ*G°*(kJ/mol)	*K*_d_(10^–6^ M)
0%	49.1 ± 2.1	18.8 ± 2.2	30.3 ± 0.6	5.6 ± 1.3
2%	49.8 ± 1.0	19.8 ± 1.1	29.9 ± 0.4	6.5 ± 0.9
6%	49.8 ± 1.1	20.2 ± 1.1	29.6 ± 0.2	7.3 ± 0.7
10%	49.4 ± 3.8	21.3 ± 4.0	28.2 ± 1.0	13 ± 5

### NMR Relaxation Dispersion Experiments Reveal the Effects of DMSO on Binding Kinetics

We used NMR spectroscopy to investigate how the binding kinetics vary with DMSO concentration. We performed ^15^N CMPG relaxation dispersion experiments at each DMSO concentration on samples that were very similar to those resulting from the ITC measurements: the protein concentration in the NMR sample was a factor of 2.7 higher than in the ITC experiments, and the protein was saturated with the ligand to approximately 95%. Under these conditions, the system is undergoing an equilibrium exchange between the free (with a relative population of *p*_F_ = 0.05) and bound (*p*_B_ = 0.95) states, which gives rise to an additional contribution to the transverse relaxation rates for those protein residues that experience different chemical shifts in the two states, see eq 3. In principle, studies of ligand binding kinetics can yield more precise rate constants if measurements be made over a wide range of *p*_B_ (*p*_F_) values. However, this is not straightforward in the case of Gal3C because this protein undergoes an intrinsic conformational exchange between the ground state and a minor, high-energy state in the absence of a ligand.^44,45^ The exchange contributions to the transverse relaxation rate constants are totally dominated by ligand binding kinetics only if *p*_B_ ≫ *p*_F_. At higher populations of the free state, the ligand binding kinetics become convolved with the intrinsic conformational exchange, which renders the analysis more intricate. For this reason, we opted to perform the present study using a single ligand/protein ratio (i.e., a single *p*_B_ value, *p*_B_ > 90%) at each DMSO concentration.

**Figure 2:**
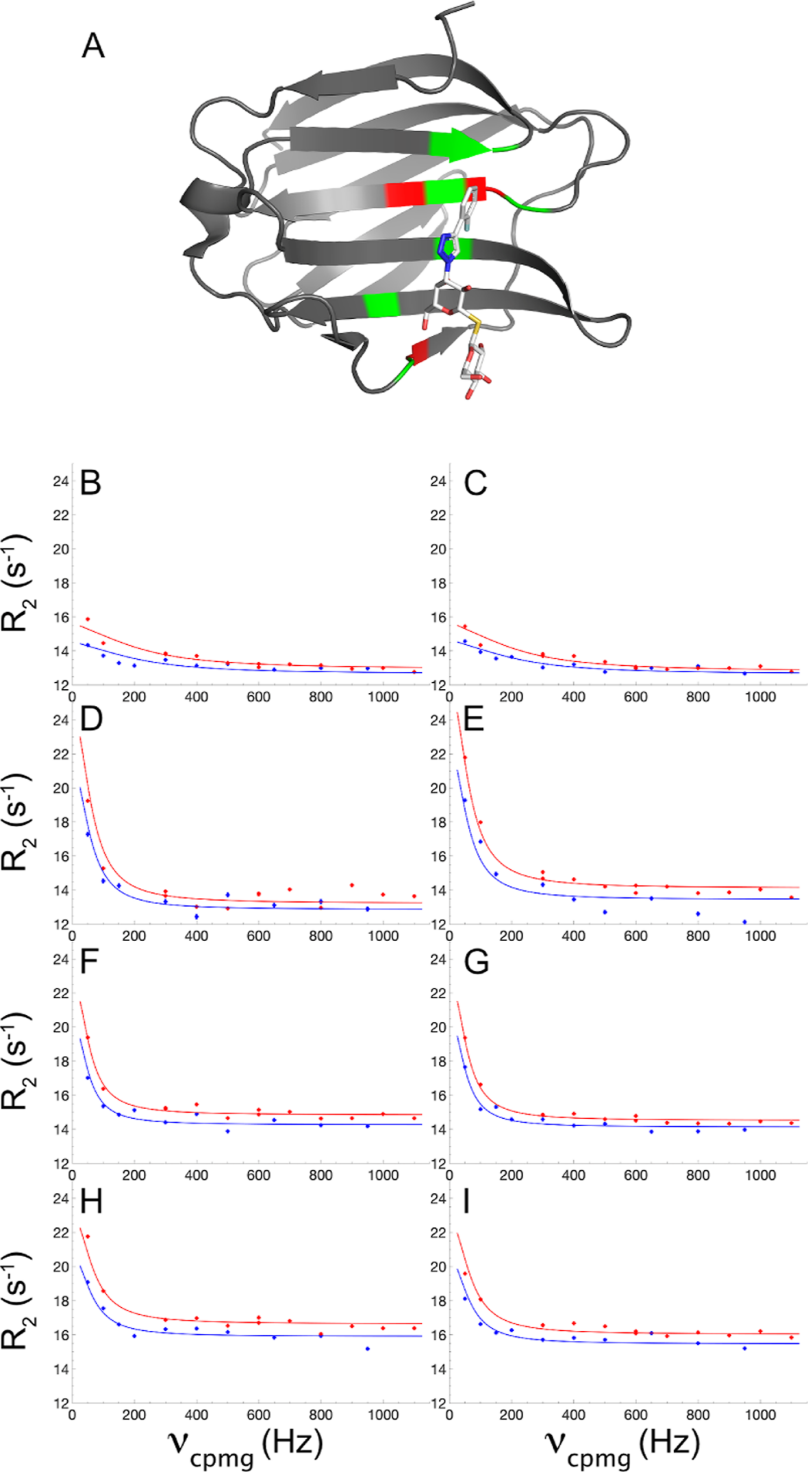
Ligand binding kinetics measured by NMR relaxation dispersion. (A) Crystal structure of the Gal3C–ligand complex with the protein and ligand shown in ribbon and stick representation, respectively. Protein residues showing significant relaxation dispersion at all the four DMSO concentrations are colored red: I145, L147, and E185. Protein residues with backbone atoms within 5 Å from the ligand are colored green. The ligand atoms are colored white (carbon), red (oxygen), blue (nitrogen), yellow (sulfur), and pale blue (fluorine). (B–I): ^15^N CPMG relaxation dispersion profiles of residues I145 (B,D,F,H) and E185 (C,E,G,I) measured in 0% DMSO (B,C), 2% DMSO (D,E), 6% DMSO (F,G), and 10% DMSO (H,I). CPMG relaxation dispersions were acquired at static magnetic field strengths of 11.7 T (blue) and 14.1 T (red). Panel A was prepared using PDB-ID: 6RZF(46) and the Pymol software package.^47^

The resulting relaxation data show significant dispersion in all the four samples for three residues: I145, L147, and E185, which are all located in or close to the binding site (Figure 2a). Several other residues also show relaxation dispersions but are partially overlapped or very broadened in one or several of the samples. Figure 2 shows relaxation dispersion data for I145 and E185, the two residues that exhibit the largest dispersion step (i.e., the largest chemical shift difference between the free and bound states). In fitting the two-state exchange model to the data, we fixed the chemical shift difference, Δω, to the value calculated from the peak positions in the HSQC spectra of the free protein and the fully saturated (*p*_B_ > 99%) protein. The population of the bound state, *p*_B_, can be calculated from *K*_d_ and the reactant concentrations in the NMR sample. However, in order to allow for minor systematic errors in these values and variation between samples, we did not only perform fits with fixed populations but also performed fits with the populations included as free parameters. We performed a global fit of the exchange data for all the three residues at each DMSO concentration; however, the fits are largely governed by I145 and E185 due to the superior data quality obtained for these residues. The gradual increase in *R*_20_, that is, the limiting value reached for high ν_cpmg_ (Figure 2), with increasing DMSO concentration is fully explained by the increase in the correlation time for rotational diffusion as a consequence of the increased viscosity of the solution.^22^

The determined exchange parameters are listed in Table 2. The global fit of CPMG data from two static magnetic field strengths and three protein residues results in relatively precise estimates of the exchange rate at each DMSO concentration. The fits including the relative population of the bound state *p*_B_ as a free parameter yield values that vary between 0.90 and 0.94 in the different samples, in good agreement with the target value of 0.95 calculated from *K*_d_ and the reactant concentrations in the NMR sample. Based on the fitted parameters *k*_ex_ and *p*_B_, we obtain *k*_off_ = *k*_ex_/(1 – *p*_B_) and then calculate *k*_on_ = *k*_off_/*K*_d_, where *K*_d_ is taken from the ITC measurements; given that the sample conditions are very nearly the same in the ITC and NMR experiments, we are confident that these values of *K*_d_ are valid.

**Table 2:** Ligand Binding Exchange Parameters

	*c*(DMSO) (v/v %)			
	0%	2%	6%	10%
Parameters (*p*_B_ Free)				
*k*_ex_ (s^–1^)	1420 ± 6	353 ± 3	276 ± 4	379 ± 2
*p*_B_^a^	0.93	0.90	0.93	0.94
*k*_off_ (s^–1^)^b^	96.6 ± 0.6	36.7 ± 0.4	18.5 ± 0.3	23.0 ± 0.1
*k*_on_(10^6^M^–1^ s^–1^)^c^	17 ± 4	5.6 ± 0.8	2.5 ± 0.3	1.8 ± 0.7
Parameters (*p*_B_ Fixed)				
*k*_ex_ (s^–1^)	855 ± 2	375 ± 7	353 ± 5	377 ± 2
*p*_B_	0.95	0.95	0.95	0.95
*k*_off_ (s^–1^)^b^	38.5 ± 0.1	18.7 ± 0.3	17.6 ± 0.2	18.8 ± 0.1
*k*_on_(10^6^M^–1^ s^–1^)^c^	7 ± 2	2.9 ± 0.4	2.4 ± 0.2	1.5 ± 0.6

a Errors in *p*_B_ are in the third decimal in all cases.

b Calculated from *k*_ex_ and *p*_F_, *k*_off_ = *k*_ex_/*p*_F_.

c Calculated from *k*_off_ and *K*_d_.

**Figure 3:**
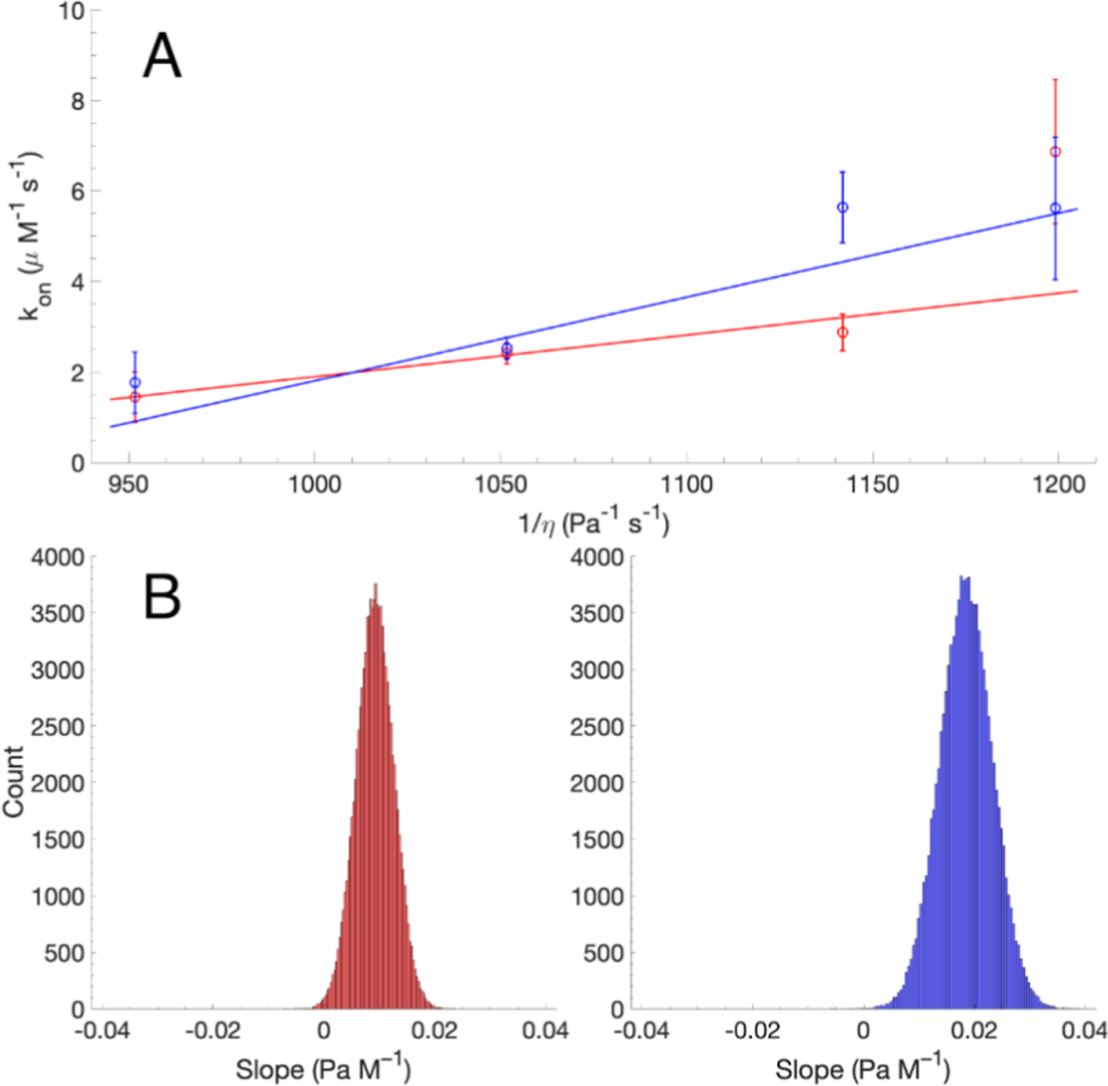
Linear regression on the effective on-rate constants (*k*_on_) plotted against the inverse dynamic viscosity (1/η) of the DMSO–water mixture. (A) *k*_on_ was determined from relaxation dispersion fits including *p*_B_ as a free parameter (blue symbols) or a fixed parameter, *p*_B_ = 0.95 (red symbols). Error bars indicate ±1 standard deviation. The blue and red lines show the fitted linear regression models. (B) Distribution of fitted values of the slope determined by linear regression on 100,000 Monte Carlo simulated data sets. Color coding as in panel A.

The two different approaches of fitting the relaxation dispersion data, viz., using *p*_B_ as a free fitting parameter or using a fixed value of *p*_B_, produce slightly different exchange rates (Table 2). Naturally, in those cases where the fitted value of *p*_B_ is similar to the calculated value, the two approaches yield similar results. The extracted value of *k*_off_ does not differ by more than a factor of 2.5 (at 0% DMSO). Similarly, *k*_on_ is broadly similar and differs at most by a factor of 2.4 (again at 0% DMSO) and is identical at the two highest DMSO concentrations. Moreover, *k*_on_ follows the same trend in both cases (Figure 3), decreasing monotonously with increasing DMSO concentration. In fact, plotting *k*_on_ versus 1/η confirms the linear relationship expected from eqs 7–9, see Figure 3.

The slope of *k*_on_ versus 1/η is (0.009 ± 0.003) Pa M^–1^ for the fit using fixed *p*_B_ and (0.020 ± 0.005) Pa M^–1^ for the free fit, which can be compared with the value expected for a diffusion-limited on-rate constant, *k*_on,D_ = 8.3 Pa M^–1^, as determined from eq 8. Thus, a significant reduction in the slope is observed compared to the diffusion-controlled case, and this conclusion holds despite the variation among the results obtained from the two different fitting protocols. The ratio *k*_on_/*k*_on,D_ provides an estimate of ρ = *k*_s_/*k*_dis_, see eq 10, which is a measure of the success rate of complex formation. The range of values determined here yields a success rate in the range ρ ≈ 0.1–0.2%. In other words, approximately 400–900 transient encounters between the ligand and protein occur on average for every successful binding event.

While it is generally difficult to calculate the effective on-rate constant from first principles, it is arguably straightforward to estimate the diffusion-controlled on-rate describing the first encounter between the ligand and protein. The simplified treatment used here neglects any effects of molecular shape by assuming that both the ligand and protein can be treated as spheres, eqs 7 and 8. Nonetheless, we believe that our approach provides a reasonable estimate of the ligand binding “success rate”, showing that several hundred binding attempts are required for each successful complex formation of Gal3C with the ligand. It should be noted that in the present case, the ligand is highly water soluble. It is possible that a more hydrophobic ligand would show less tendency to dissociate from the protein surface and therefore exhibit a greater “success rate” than what is observed here. Similarly, electrostatic interactions between the ligand and protein—which do not play any significant role in the present case since the ligand is uncharged—should result in an improved success rate.^41^ We expect that the ligand-binding success rate is highly system dependent.

### Concluding Remarks

We conclude that the addition of up to 10% DMSO does not affect the binding thermodynamics to any appreciable extent in the studied model system. While there is a subtle trend toward decreasing binding affinity (i.e., increasing *K*_d_) with increasing DMSO concentration, the effect is within a factor of 2, which translates into a few kJ/mol in standard free energy. The subtle change in the standard free energy of binding originates entirely from an entropic effect, whereas the enthalpy of binding does not vary with DMSO concentration, which is expected since DMSO does not inhibit the binding site of galectin-3.^22^ However, it is entirely possible that DMSO might act as a competitive inhibitor in other systems.

Our study reveals a statistically significant effect on the effective on-rate constant of binding, which decreases with increasing DMSO concentration. This effect is expected from the increase in viscosity with increasing DMSO concentration, which acts to decrease the diffusion coefficient. The slope of *k*_on_ versus 1/η is significantly reduced from the value expected for a diffusion-controlled reaction, indicating that several hundred protein–ligand encounters occur for each productive binding event. In other words, each diffusive encounter between the ligand and protein has less than 1% chance of resulting in a productive complex before the encounter complex dissociates. Kinetic solvent viscosity effects have been used previously to provide detailed information on the reaction steps of enzyme catalysis.^48^ NMR spectroscopy enables high-resolution mapping of protein–ligand binding, which has the advantage to identify binding specifically to the binding site, allowing for comparison of the effective association rate with the diffusion-controlled rate of encounters. We anticipate that the present approach should be widely applicable—by a judicious choice of viscogens suitable for each specific protein–ligand system—to studies of protein–ligand binding kinetics and serve as a useful complement to methods for characterizing encounter complexes.^49,50^

## Supporting Information Available

ITC binding isotherms (PDF)

## Abbreviations

CPMG: Carr–Purcell–Meiboom–Gill
DMSO: dimethyl sulfoxide
EDTA: ethylenediaminetetraacetic acid
Gal3C: the carbohydrate binding domain of galectin-3
HEPES: 4-(2-hydroxyethyl)-1-piperazinethanesulfonic acid
HSQC: heteronuclear single-quantum coherence
ITC: isothermal titration calorimetry
TCEP: tris(2-carboxyethyl)phosphine hydrochloride
