# Supplementary material for: Cosolvent Dimethyl Sulfoxide Influences Protein–Ligand Binding Kinetics via Solvent Viscosity Effects: Revealing the Success Rate of Complex Formation Following Diffusive Protein–Ligand Encounter
Source: Biochemistry. 2022 Dec 21;62(1):44–52. doi: 10.1021/acs.biochem.2c00507 (PMC9813907; doi:10.1021/acs.biochem.2c00507)
Supplement: Supplementary file 1 — bi2c00507_si_001.pdf [file bi2c00507_si_001.pdf]

Supplementary Information

The cosolvent dimethylsulfoxide influences protein–ligand binding kinetics via solvent viscosity effects: Revealing the success rate of complex formation following diffusive protein–ligand encounter

Sven Wernersson, Simon Birgersson, Mikael Akke*

Division of Biophysical Chemistry, Center for Molecular Protein Science, Department of Chemistry, Lund University, P. O. Box 124, SE-221 00 Lund, Sweden

* Corresponding author: mikael.akke@bpc.lu.se

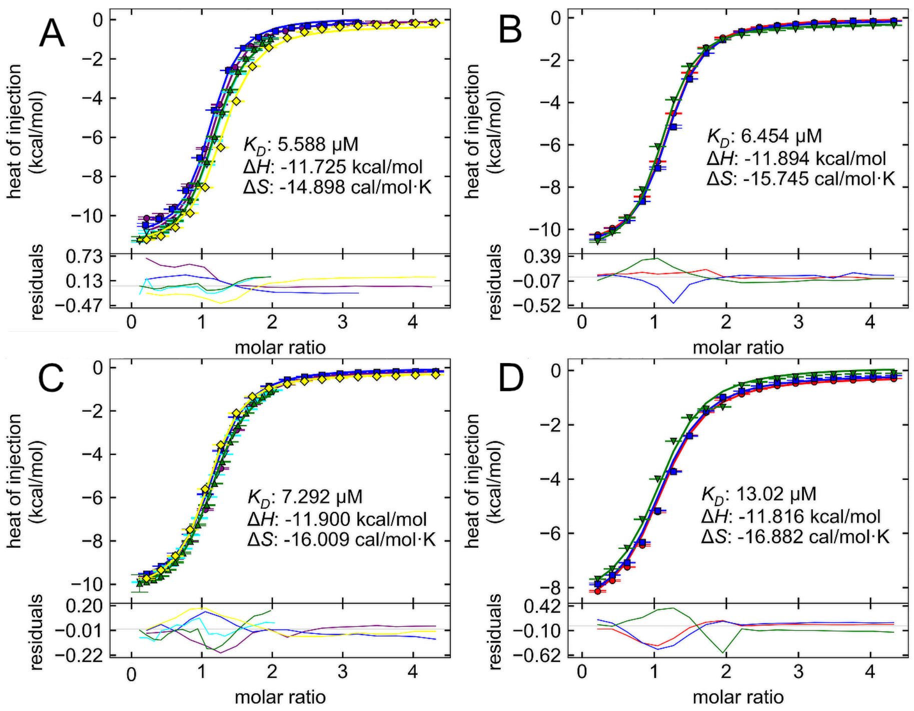

Figure S1. Superimposed isotherms from triplicate isothermal titration calorimetry measurements of protein ligand binding. (A) 0% DMSO, (B) 2% DMSO, (C) 6% DMSO, (D) 10% DMSO.
